# Diagnostic levels of C-reactive protein (CRP), epinephrine (EP), cortisol (COR), and norepinephrine (NE) in patients undergoing laparoscopic surgery for gallstones receiving anti-inflammatory therapy

**DOI:** 10.5937/jomb0-54684

**Published:** 2025-09-05

**Authors:** Gaoqiang Wang

**Affiliations:** 1 Zhengzhou Shuqing Medical College, Department of Clinical Medicine, Zhengzhou, 450000, China

**Keywords:** gallstones, laparoscopy, anti-inflammatory bile soup, C-reactive protein, epinephrine, cortisol, norepinephrine, kamen u žučnoj kesi, laparoskopksa hirurgija, antiinflamatorna "supa" za žučnu kesu, C-reaktivni protein, epinefrin, kortizol, norepinefrin

## Abstract

**Background:**

The study aimed to investigate the effect of anti-inflammatory and choleretic soup on CRP, Ep, Cor, NE, and gallbladder function recovery in patients following laparoscopic cholecystectomy.

**Methods:**

A total of 111 patients with gallstones who underwent laparoscopic surgery at the hospital between November 2019 and March 2024 were selected. All patients were randomly assigned to two groups using a random number method. The control group (n=56) received extracorporeal circulation treatment alone, while the experimental group (n=55) received additional treatment with anti-inflammatory bile soup. Differences in the levels of C-reactive protein (CRP), epinephrine (Ep), cortisol (Cor), and norepinephrine (NE) were compared and analysed between the two groups. The effects on gallbladder function recovery were also evaluated.

**Results:**

Three months post-treatment, the levels of CRP, Ep, Cor, and NE in both groups had decreased compared to pre-treatment levels. Post-treatment levels of these indices were significantly lower in the experimental group than in the control group (P<0.05). Furthermore, the gallbladder wall thickness and fasting gallbladder volume (FV) in both groups were reduced compared to pre-treatment levels, while the gallbladder contraction rate improved. The experimental group exhibited better gallbladder function outcomes across various indices than the control group (P<0.05).

**Conclusions:**

The application of anti-inflammatory and choleretic soup in patients following laparoscopic cholecystectomy contributes to improvements in physiological parameters, enhances the recovery of gallbladder functional indices, and improves the quality of life during prognosis and rehabilitation.

## Abstract (sr)

**Uvod:**

Istraživanje je imalo za cilj ispitivanje efekta antiinflamatorne i holeretičke "supe" na CRP, Ep, Cor, NE i oporavak funkcije žučne kese kod pacijenata nakon laparoskopskih holecistektomija.

**Metode:**

Za istraživanje je odabrano ukupno 111 pacijenata sa kamenjem u žučnoj kesi koji su podvrgnuti laparoskopskoj operaciji u bolnici između novembra 2019. i marta 2024. godine. Svi pacijenti su nasumično raspoređeni u dve grupe korišćenjem metode nasumičnog broja. Kontrolna grupa (n=56) je primala samo tretman ekstrakorporalne cirkulacije, dok je eksperimentalna grupa (n=55) primala dodatni tretman antiinflamatornom "supom". Upoređivane su i analizirane razlike u nivoima C-reaktivnog proteina (CRP), epinefrina (Ep), kortizola (Cor) i norepinefrina (NE) između dve grupe. Takođe, ocenjeni su i efekti na oporavak funkcije žučne kese.

**Rezultati:**

Tri meseca nakon tretmana, nivoi CRP, Ep, Cor i NE u obe grupe su opali u poređenju sa nivoima pre tretmana. Nivoi ovih indeksa posle tretmana su bili značajno niži u eksperimentalnoj nego u kontrolnoj grupi (P<0,05). Takođe, debljina zida i volumen žučne kese u stanju gladovanja (FV) u obe grupe su smanjeni u poređenju sa nivoima pre tretmana, dok je stopa kontrakcije žučne kese poboljšana. Eksperimentalna grupa je pokazala bolje rezultate u funkciji žučne kese u različitim indeksima u odnosu na kontrolnu grupu (P<0,05).

**Zaključak:**

Primena antiinflamatorne i holeretičke "supe" kod pacijenata nakon laparoskopskih holecistektomija doprinosi poboljšanju fizioloških parametara, unapređuje oporavak funkcionalnih indeksa žučne kese i poboljšava kvalitet života tokom perioda prognoze i rehabilitacije.

## Introduction

Gallstones are a common condition of the biliary system and are highly prevalent in the Asia- Pacific region. The deterioration of gallstones can lead to the development of more severe cholangitis, or worse, cholangitis patients can progressively deteriorate into cholangiocarcinoma. The presence of gallstones can lead to compression of the accompanying portal vein, which can reduce blood flow and jeopardise the patient’s life. From the current clinical treatment status, it is mainly treated by surgery, and long-term clinical treatment found that surgical treatment is still the most effective program [1] [2]. With the continuous development of medical technology, many clinical operations began to combine laparoscopic cholecystectomy to improve the clinical treatment effect of gallstones further. The main reason is that laparoscopic cholecystectomy brings minimal trauma and a low incidence of complications. In addition, because the majority of patients with gallstones develop stones that can lead to bile duct infection, leading to inflammatory stenosis and obstruction of proximal bile duct expansion, surgical treatment still carries certain safety risks [3] [4] [5].

In recent years, with the widespread application of traditional Chinese medicine (TCM) as an adjunctive therapy in clinical diagnosis and treatment, research has shown that the success rate and prognosis of laparoscopic resection significantly improve when combined with TCM therapy. In laparoscopic surgery for gallstones, more attending physicians opt to incorporate TCM therapies to reduce surgical risks and enhance patient prognosis and recovery outcomes [3] [6] [7].

Anti-inflammatory and choleretic soup (AI-CS) is a Chinese herbal preparation composed of several herbs known for its effectiveness in promoting blood circulation, resolving blood stasis, reducing inflammation, alleviating pain, and regulating liver qi. AI-CS is increasingly being applied in laparoscopic surgery for gallstones. However, its adoption remains limited, as its clinical adjuvant therapeutic mechanisms are not yet fully understood, and existing studies have not explored these mechanisms in depth [8] [9] [10].

To further analyse the clinical efficacy of laparoscopic surgery combined with AI-CS, this study examined changes in multiple patient indicators before and after treatment (Bt & At), such as C-reactive protein (CRP), epinephrine (Ep), cortisol (Cor), and norepinephrine (NE). The goal is to clarify the mechanism of the combined treatment and provide theoretical support for improving patients’ gallbladder function recovery.

## Materials and methods

### General information

This study selected 111 patients with gallstones who underwent laparoscopic surgery at the hospital between November 2019 and March 2024 as study participants. The control group (C) consisted of 56 patients who received conventional treatment alone, while the experimental group (E) included 55 patients who received a combined application of AI-CS in addition to conventional treatment. The random number method (RNM) assigned the participants to the groups. There were no statistically significant differences among the patients in terms of age, gender, type of stone, body mass index (BMI), disease duration, or type of underlying condition (*P*>0.05).

Inclusion criteria: (1) Meeting the relevant diagnostic criteria for gallbladder stones as outlined in the 2014 Chinese Consensus on Internal Medicine Diagnosis and Treatment of Chronic Cholecystitis and Gallstones; (2) Physically meeting the criteria and requirements for laparoscopic surgical treatment and undergoing such surgery for the first time; (3) Presence of bacterial infections; (4) Stable postoperative vital signs; and (5) Demonstrating good reading, writing, cognitive, and mental health. (6) Patients and their families signed an informed consent form.

Exclusion criteria: (1) History of previous upper abdominal surgery; (2) Presence of bile ducttumours, stenosis of the lower end of the common bile duct, severe cholangitis, or severe cholecystitis; (3) Concomitant pancreatic enlargement and dilation; (4) Receipt of cholagogic or anti-infective treatment within the past two weeks; (5) Presence of cardiovascular, cerebrovascular, haematological, or immune system diseases.

### Research methodology

### Grouping method

This study randomly segments the 111 patients into 2 groups using RNM (11), i.e. C (n=56) and E (n=55).

### Surgical method

Both groups of patients underwent laparoscopic surgery. Following routine anaesthesia and thorough preoperative preparation, a 20–22 mm incision was created along the upper edge of the umbilicus. A curved incision was subsequently made on the inner side of this site, carefully dissecting the skin and tissue layers in sequence. Pneumoperitoneum was established near the navel, with carbon dioxide pressure maintained at 10–20 mmHg. A 10 mm Trocar was inserted into the primary incision, followed by two additional 5 mm Trocar incisions on either side, forming an inverted triangular arrangement.

laparoscope was introduced through the observation port to inspect the gallbladder tissue within the abdominal cavity [11] [12] [13] [14]. Electric hooks and traction forceps were positioned on either side of the operative field to apply traction to the gallbladder floor. The gallbladder duct and artery were fully mobilised, and gallbladder duct forceps were employed to carefully dissect and sever the gallbladder tissue 0.5 cm from the common bile duct.

The affected area was excised, and meticulous suturing was performed. The Trocars were then removed, and the tissue and skin layers were sequentially closed with sutures.

### Therapeutic method

C: C’s patients received somatic peptide therapy during the perioperative period. The initial treatment dose for the patient was 250 mg, and intravenous infusion was used. After 3 consecutive days of treatment, the dosage was adjusted to 3mg and intravenous infusion was performed at 250 mg per hour.

E: Based on C’s treatment, add AI-CS and take warm water twice in the morning and evening. The total cure period for both E and C patients is 4 weeks.

### Observation indicators

Firstly, the patient’s stress response during treatment will be evaluated. A 5 mL fasting blood sample will be collected from the patient’s elbow vein before treatment and one month afterwards. A serum will be obtained through centrifugation and temporarily stored in a freezer at -80°C for later analysis.

Using immune transmission turbidimetry, the experiment utilised the Mindray BS-280 fully automated biochemical analyser to measure C-reactive protein (CRP) levels. High-performance liquid chromatography was employed to detect epinephrine (Ep) and norepinephrine (NE) levels, while cortisol (Cor) levels were determined using radioimmunoassay.

The same physician must adhere strictly to the instructions for multi-indicator testing and analysis throughout the testing process to ensure consistency and accuracy [15] [16].

Secondly, the patient’s gallbladder function should be evaluated before and after treatment. Patients should be instructed to fast for 6 hours before treatment and for 1 month afterwards. After fasting, the thickness of the gallbladder wall should be measured using an ultrasound diagnostic instrument. Additionally, the maximum longitudinal diameter, maximum transverse diameter, and anteroposterior diameter of the gallbladder in the fasting state should be measured to calculate the fasting gallbladder volume (FV) [17] [18].

Following the fasting measurements, the patient may be given a fat-rich meal to stimulate gallbladder contraction. After the meal, the gallbladder’s longitudinal, transverse, and anteroposterior diameters should be measured again to calculate the residual gallbladder volume (RV). The gallbladder contraction rate should then be determined based on the fasting gallbladder volume and the residual volume.

The treatment effect is evaluated by assessing the patient’s levels of inflammatory factors, the occurrence of complications (CPO), and their quality of life. Serum procalcitonin (PCT), interleukin-17 (IL-17), and interleukin-22 (IL-22) were selected as indicators for evaluating inflammatory factors. The assessment of CPO primarily requires physicians and nurses to monitor the patient during and after treatment for complications such as infection, abdominal distension, or common bile duct injury [19] [20].

Evaluating the patient’s quality of life involves a responsible nurse assessing the gastrointestinal quality of life index (GIQLI) before and after treatment. The GIQLI scale encompasses four dimensions: subjective symptoms, physiological functional status, social activity status, and psychological status. The total score is 144 points, with higher scores indicating better quality of life.

### Statistical methods

The data were analysed using SPSS 26.0 software, and the number of cases (n) and rate (%) were used to describe the count data, and the ^2^ test was performed. The mean standard deviation (x±s) was taken to describe normally distributed measurement data and a t-test was performed. All data difference analyses were bounded by *P*=0.05 when* P*<0.05 indicated a significant difference.

## Results

### Comparison of general information

The general characteristics of the patients participating in the experiment, including age, gender, stone type, BMI, disease duration, and type of underlying conditions, are presented in Table 1. Table 1 indicates that the evaluation indicators for the two groups of patients have a P-value greater than 0.05 (*P*>0.05), suggesting that a comparative analysis between the two groups is valid.

**Table 1:** Two sets of baseline data.

Evaluating indicator		C	E	*P*
Gender	male	31	30	>0.05
	female	25	25	
Age		52.48±4.16	52.67±4.09	>0.05
Stone type	Primary choledocholithiasis	30	31	>0.05
	Gallbladder stones combined with<br>secondary common bile duct stones	26	24	
BMI (kg/m^2^)		22.73±2.30	22.71±2.11	>0.05
Course of Disease (Year)		5.16±1.62	5.27±1.15	>0.05
Merge underlying<br>diseases	hypertension	21	20	>0.05
	diabetes	29	29	
	hyperlipidemia	6	6	

### Treatment risk analysis

The univariate analysis of patient data obtained from laparoscopic cholecystectomy, using group C patients as the study sample, is presented in Table 2. In Table 2, a P-value of less than 0.05 (*P*<0.05) indicates that a factor is a risk factor. The single-factor analysis of all indicators identified eight risk factors affecting surgical risk, while the P-values for the remaining indicators were more significant than 0.05.

**Table 2:** Intraoperative risk single factor analysis.

Indicator (n=267)	P	OR	95%CI
Gender (male/female)	0.801	1.725	(0.923~2.004)
Age (>60 years old/ 60 years old)	0.109	2.031	(1.573~2.361)
Previous surgical history (none/present)	0.047	1.543	(1.123~1.834)
Recurrent abdominal pain (no/with)	0.017	1.957	(1.403~2.117)
Red blood cell count (normal/abnormal)	0.161	2.369	(2.134~2.578)
Platelet count (normal/abnormal)	0.662	1.180	(0.921~1.665)
Lymphocyte percentage (normal/abnormal)	0.049	3.405	(3.054~3.723)
Common bile duct stones (no/with)	0.038	1.113	(0.623~1.527)
Stone type (simple/complex)	0.045	2.264	(2.004~2.517)
Biliary stenosis (no/with)	0.015	4.184	(3.463~4.326)
Anesthesia score ( 2 points/>2 points)	0.026	3.729	(3.575~3.964)
Resection range	0.007	6.557	(5.773~7.016)

Table 3 shows that the eight risk factors identified through single-factor analysis were subjected to factor analysis to determine the independent risk factors for surgical risk. Table 3 presents five independent risk factors for laparoscopic cholecystectomy: recurrent abdominal pain, common bile duct stones, bile duct stenosis, anaesthesia score, and resection range.

**Table 3:** Intraoperative risk multifactor analysis.

Indicator (n=267)	P	OR	95%CI
Recurrent<br>abdominal pain	0.024	2.951	(1.174~4.106)
Choledocholithiasis	0.010	3.645	(1.348~5.783)
Biliary Stricture	0.009	3.672	(1.987~6.873)
Anesthesia score	0.026	3.745	(1.870~5.787)
Resection range	0.007	3.482	(1.531~5.284)

### Safety evaluation of treatment plans

**Figure 1:**
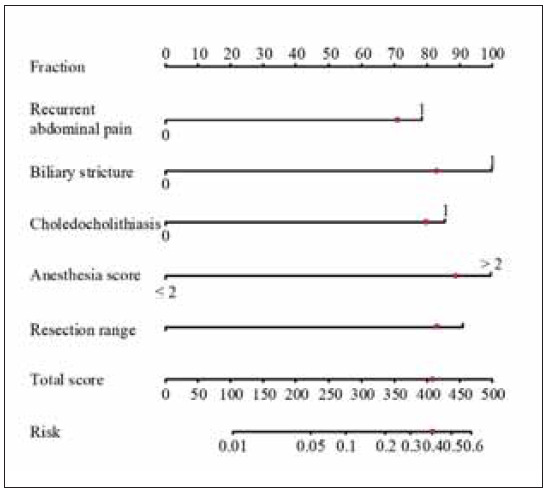
Prediction of intraoperative risk column chart.

Construct a column chart for risk prediction in laparoscopic cholecystectomy based on the five independent risk factors identified, as shown in Figure 1. From Figure 1, it can be concluded that, based on the risk factors derived from the patient’s examination, the highest intraoperative risk for the patient may reach 40%.

**Figure 2:**
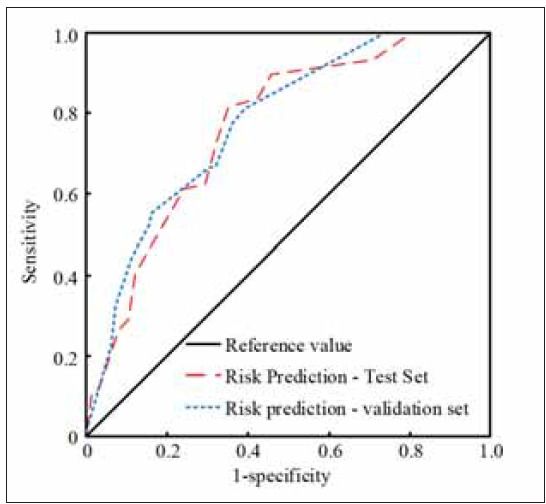
Column chart predictive roc curve.

To verify the performance of the column chart in risk prediction, the ROC curve was used to assess its predictive discrimination, and the clinical data of C were divided into a test set and a validation set. The results are shown in Figure 2. Figure 2 demonstrates that the area under the ROC curve predicted by the column chart in the test set was 0.784 (95% CI, 0.725–0.881). In the validation set, the area under the ROC curve predicted by the column chart for risk was 0.749 (95% CI, 0.672–0.911).

**Figure 3:**
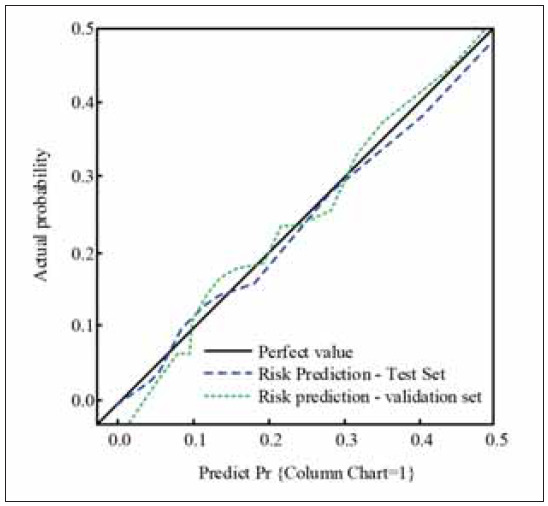
Analysis of fitting of intraoperative risk prediction.

Figure 3 uses calibration curve analysis to assess the fit of the column chart in risk prediction applications. In the test set of Figure 3, there is a slight difference between the predicted risk and the ideal value using the column chart technique. When comparing the predicted intraoperative risk in the validation set with the observed intraoperative risk, P = 0.429. In the validation set, the risk prediction curve of the column chart is relatively close to the ideal value, with a P value of 0.673 when compared to the actual results. No statistically significant difference was found between the two datasets.

**Figure 4:**
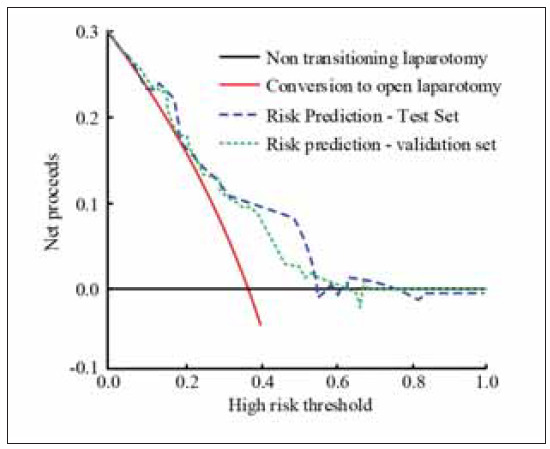
Analysis of clinical decision curve for risk prediction using a column chart.

Figure 4 uses clinical decision curves to analyse the clinical value of intraoperative risk prediction. The test set in Figure 4 shows that after the high-risk threshold reaches 0.57, the net benefit of the risk prediction model falls below 0 for the first time. In the validation set, after the high-risk threshold reaches 0.63, the net benefit decreases to 0. In summary, the surgical protocol used in this study demonstrates a lower risk.

### Changes in CRP, Ep, Cor, NE indicators in patients Bt&At

Table 4 shows the changes and differences in CRP, Ep, Cor, and NE levels between Bt and At in the two sets. The CRP, Ep, Cor, and NE levels in patients between Bt and At showed significant changes, with all indicators being lower after treatment than before. This indicates that, under different treatment regimens, both patient groups received effective treatment. Additionally, there were significant differences in various E indicators after treatment compared to C (*P*<0.05).

**Table 4:** Analysis of differences in CRP, Ep, Cor, NE indicators.

Group	Cases	CRP (mg/L)		Ep (ng/mL)		Cor (ng/mL)		NE (μg/L)	
		Pre-treatment	After<br>treatment	Pre-treatment	After<br>treatment	Pre-treatment	After<br>treatment	Pre-treatment	After<br>treatment
C	56	27.46±5.37	14.88±4.07	0.43±0.008	0.39±0.06	158.32±20.60	141.77±15.89	211.10±13.55	62.78±4.26
E	55	27.52±5.33	9.57±3.14	0.44±0.009	0.21±0.03	158.43±20.57	120.36±13.91	210.64±13.48	30.19±2.51
*t*		0.187	2.355	0.018	3.340	0.039	10.172	0.014	37.451
*P*		0.852	0.020	0.986	0.001	0.642	0.004	0.269	0.001

### Comparison of gallbladder function Bt&At

Table 5 shows the results of the analysis of gallbladder function differences between Bt and At in patients. This study evaluated gallbladder function using gallbladder wall thickness, FV, and the gallbladder contraction rate and analysed the recovery of gallbladder function in both patient groups. Table 5 demonstrates the differences in gallbladder wall thickness, FV, and gallbladder contraction rate between patients Bt and At. The thickness of the gallbladder wall and FV were reduced post-treatment, while the gallbladder contraction rate increased (*P*<0.05). When comparing indicators between the two patient groups, significant differences were found in variousindicators after treatment (*P*<0.05).

**Table 5:** Analysis of differences in gallbladder function.

Group	n	Gallbladder wall thickness (mm)		FV (mm^3^)		Gallbladder contraction rate (%)	
		Pre-treatment	After treatment	Pre-treatment	After treatment	Pre-treatment	After treatment
C	56	5.54±1.26	3.75±0.85	28.51±3.04	24.63±5.07	56.26±9.16	62.07±4.39
E	55	5.49±1.20	2.61±0.64	28.59±3.07	20.31±4.94	56.33±9.09	71.42±3.98
*t*		0.065	2.903	0.042	2.538	0.054	3.314
*P*		0.948	0.005	0.967	0.013	0.957	0.001

### Comparison of serum inflammatory factors Bt&At

Table 6 shows the comparison results of the differences in inflammatory factors. This study usedPCT, IL-17, and IL-22 as evaluation indicators. The differences in various indicators between Bt and At are significant, with the levels of inflammatory factors showing a downward trend in both groups. No significant distinction was found in the various indicators before treatment when comparing the differences between C and E. However, after treatment, a significant difference in the levels of inflammatory factors was observed (*P*<0.05).

**Table 6:** Analysis of differences in serum inflammatory factors Bt&At.

Group	n	PCT (ng/mL)		IL-17 (mg/L)		IL-22 (ng/mL)	
		Pre-treatment	After treatment	Pre-treatment	After treatment	Pre-treatment	After treatment
C	56	6.12±10.37	3.29±0.69	30.37±5.27	26.48±3.33	50.54±10.12	35.41±7.29
E	55	6.19±1.14	1.48±0.55	30.44±5.06	23.47±4.04	50.57±10.14	26.55±4.36
*t*		0.115	3.456	0.024	6.138	0.019	4.453
*P*		0.784	0.020	0.867	0.001	0.974	0.004

### Correlation analysis between gallbladder contraction rate and various indicators

**Figure 5:**
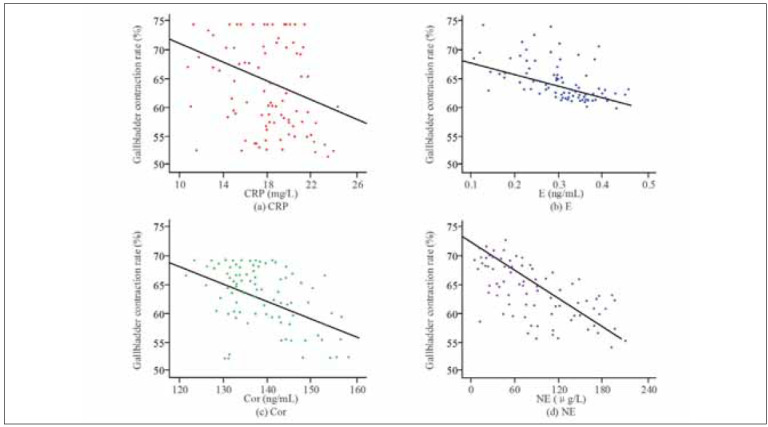
Correlation analysis between gallbladder contraction rate and CRP, Ep, Cor, NE.

This study used the gallbladder contraction rate to evaluate the correlation between various indicators in patients with gallstones to evaluate gallbladder function recovery. A correlation analysis was performed using Pearson’s correlation coefficient, and the correlation curve between the gallbladder contraction rate and CRP, Ep, Cor, and NE was plotted, as shown in Figure 5. Figure 5 demonstrates a significant correlation between the gallbladder contraction rate of patients and CRP, Ep, Cor, and NE. As the level of each indicator increases, the patients’ gallbladder contraction rate shows a continuously decreasing trend.

**Figure 6:**
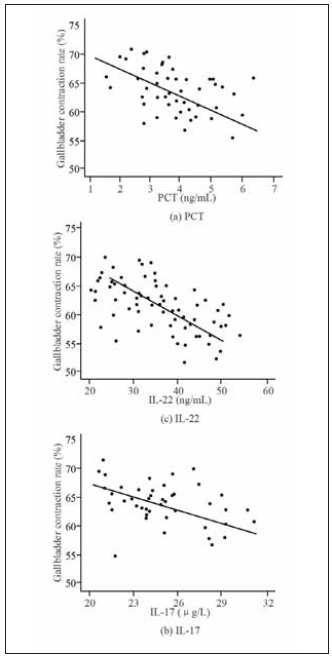
Correlation analysis between gallbladder contraction rate and PCT, IL-17, IL-22.

Secondly, a Pearson correlation analysis was performed to analyse the relationship between the gallbladder contraction rate and the three indicators of PCT, IL-17, and IL-22, as shown in Figure 6. In Figure 6, the gallbladder contraction rate of the patient is correlated with PCT, IL-17, and IL-22, showing a negative correlation. As the levels of the various indicators decrease, the gallbladder contraction rate of patients increases.

### Comparison of GIQLI scale scores Bt&At

Table 7 shows the GIQLI scale scores of patients Bt&At. Table 7 shows the four evaluation indicators of conscious symptoms, physiological function status, social activity status, and psychological status of patients with C and E were significantly higher after treatment. In addition, the differences in scores of various indicators after treatment were compared. It can be observed that after treatment, there is an obvious distinction in the scores, and the scores of all E indicators are higher than those of C (*P*<0.05).

**Table 7:** GIQLI scale scores Bt&At (x̅ ± s, grade).

Group	n	Subjective symptoms		Physiological functional<br>status		Social activity status		Psychology	
		Pre-treatment	After<br>treatment	Pre-treatment	After<br>treatment	Pre-treatment	After<br>treatment	Pre-treatment	After<br>treatment
C	56	46.43±5.21	65.14±6.15	10.24±1.53	19.44±2.68	8.67±2.16	12.52±2.87	7.55±2.36	13.24±3.12
E	55	46.74±5.11	70.14±7.62	10.17±1.49	22.16±3.05	8.81±2.03	15.48±3.16	7.56±2.71	15.48±3.37
*t*		0.235	3.606	0.221	4.896	0.378	4.786	0.015	3.412
*P*		0.844	0.001	0.883	0.000	0.731	0.000	0.907	0.001

### Prognostic analysis of AI-CS combined with laparoscopic surgery for patients with gallstones

**Figure 7:**
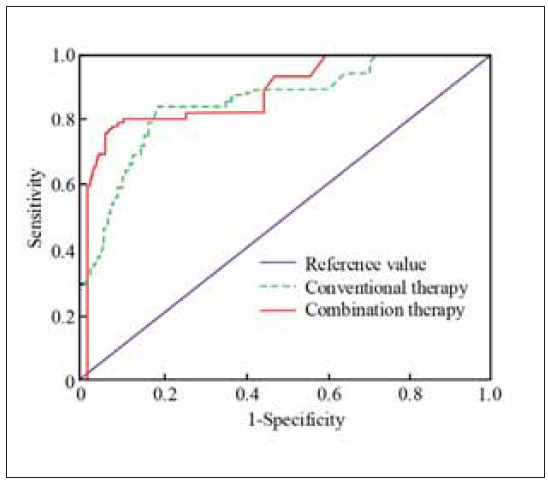
ROC analysis of the efficacy of different treatment methods.

Figure 7 shows the comparative analysis results of the subject work curve (ROC) between the combination of AI-CS and laparoscopic surgery with conventional treatment regimens. The purpose is to determine the feasibility of different treatment methods through ROC curves. The outcomes validated that the area under the ROC of the AI-CS combined with laparoscopic surgery used in the study was 0.849. The area below the ROC for conventional treatment regimens is 0.797. Comparing the area under the curve of the two treatment regimens, it was found that the area under the curve of the combined cure was significantly higher than the conventional treatment regimen (*P*<0.05).

Table 8 shows the analysis results of the incidence of complications in two groups of patients. The incidence of complications in patients with E was significantly higher than in C (*P*<0.05).

**Table 8:** Comparative data of complications.

Group	Infection	Abdominal<br>distension	Common<br>bile duct<br>injury	Incidence of<br>complications
C	4	3	0	12.5%
E	1	1	0	3.6%
χ^2^				4.170
*P*				0.001

**Figure 8:**
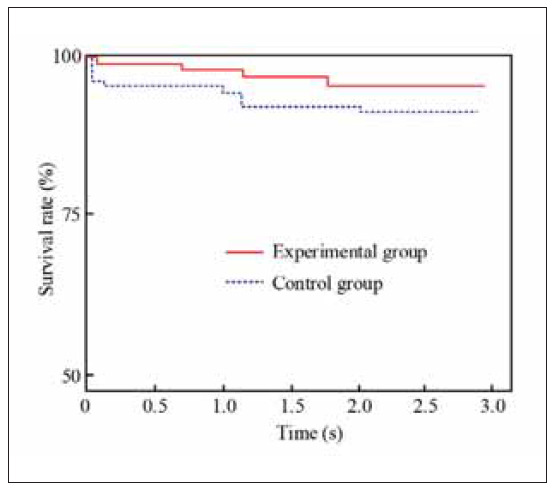
Analysis of survival curves for two groups of patients.

Finally, the overall prognosis of patients with E and C was analysed through survival analysis, as shown in Figure 8. From Figure 8, the overall survival rate of patients with E three months after treatment reached 95%, while the survival rate of patients with C three months after treatment was over 90%. Overall, the prognosis of patients with E is better.

## Discussion

The study demonstrates that while baseline characteristics between the two groups were comparable (*P*>0.05), treatment risk analysis identified five independent risk factors for laparoscopic cholecystectomy. The AI-CS combined approach improved clinical outcomes significantly, including better gallbladder function recovery, reduced inflammation, and higher GIQLI scores compared to conventional methods (*P*<0.05). Prognostic analysis further confirmed the superiority of the AI-CS combination, showing lower complication rates, higher survival rates, and improved risk prediction with an area under the ROC curve of 0.849 compared to 0.797 for standard treatments.

### Analysis of the significance of using AI-CS for postoperative treatment of laparoscopic gallstones

With the continuous advancement of science and technology, clinical medicine has embraced newdevelopment directions. In this process, laparoscopy has gradually become a widely used auxiliary tool in clinical medicine, aimed at optimising surgical procedures, improving surgical success rates, and reducing the incidence of complications in patients [21] [22] [23].

In the treatment of gallstones, the application of laparoscopy has become increasingly common. The primary advantage of laparoscopy is its ability to facilitate minimally invasive surgery with minimal bleeding. For patients with gallstones, laparoscopic surgery offers a superior postoperative experience. However, gallstones are often associated with gallbladder inflammation, which is a prevalent condition. This inflammation can lead to tissue adhesions, which exacerbate the condition and complicate the management of gallstones. Such circumstances reduce the patient’s quality of life and hinder the successful execution of laparoscopic surgery [24] [25].

Research has demonstrated that anti-inflammatory choleretic soup (AI-CS) can effectively alleviate inflammation in patients. Clinical practice has shown that using AI-CS yields significant benefits in managing gallstones. However, AI-CS alone is insufficient for effectively treating gallstones, particularly in patients with more severe cholecystitis, whose impact is limited [26].

To address this, the present study aims to combine laparoscopic surgery with AI-CS. The objective is to enhance the effectiveness of laparoscopic surgery by using AI-CS to treat inflammation, thereby providing a theoretical foundation for the clinical management of gallstone patients. This approach offers scientific and practical treatment measures to improve gallstone patients’ prognosis and quality of care.

### The effect of AI-CS on CRP, E, Cor, NE in patients undergoing laparoscopic gallstone surgery

CRP, Ep, Cor, and NE are key indicators of a patient’s stress response and are commonly used to evaluate the effectiveness of treatments for gallstone patients. This study assessed the changes in these markers to evaluate the therapeutic impact of AI-CS combined with laparoscopic surgery for gallstones. Results showed a consistent decline in CRP, Ep, Cor, and NE levels in all patients, indicating a progressive reduction in stress response under routine and combination therapy. Notably, the post-treatment levels of these markers were significantly lower in the AI-CS group (E) compared to the control group (C), with all differences reaching statistical significance.

Previous studies have reported that traditional Chinese medicine decoctions can improve stress response markers in gallstone patients, with some directly highlighting the role of AI-CS in reducing CRP, Ep, Cor, and NE levels [27]. These findings align with this study’s results, reinforcing the conclusion that AI-CS positively impacts laparoscopic surgery outcomes for gallstone patients. These results underscore the clinical value of AI-CS as an effective adjunct in treating gallstones.

### The effect of AI-CS on inflammatory factors in patients undergoing laparoscopic gallstone surgery

Inflammation is a significant challenge in laparoscopic surgery for gallstones, and reducing it is critical to improving surgical success rates. This study integrated AI-CS to enhance treatment outcomes for gallstone patients and utilised PCT, IL-17, and IL-22 as inflammatory markers for evaluating efficacy. The results demonstrated a consistent decline in PCT, IL-17, and IL-22 expression levels across all patients. Intergroup comparisons revealed that these markers decreased significantly in the AI-CS group (E) than in the control group (C), indicating lower post-treatment levels in the E group.

Previous research has highlighted the primary function of AI-CS in mitigating inflammatory responses. Specifically, in treating gallstones, AI-CS enables targeted intervention at the site of gallbladder inflammation. The findings of this study corroborate these earlier results, further validating the efficacy of AI-CS in reducing inflammation and enhancing treatment outcomes for gallstone patients [28].

### The effect of AI-CS on the recovery of gallbladder function in patients undergoing laparoscopic gallstone surgery

Gallstones significantly impair normal gallbladder function, making timely treatment through laparoscopic surgery critical. This study assessed the impact of AI-CS on gallbladder function recovery in patients undergoing laparoscopic surgery for gallstones. Results indicated that patients receiving combination therapy (group E) experienced a significant reduction in gallbladder wall thickness and FV post-treatment, with markedly lower levels observed in the control group (C). Additionally, the gallbladder contraction rate in group E was significantly higher after treatment compared to group C.

These findings align with previous research showing a notable decrease in gallbladder wall thickness and a gradual normalisation of gallbladder contraction rates following laparoscopic surgery for gallstones [29]. In this study, all patients showed improvements, with group E exhibiting a more pronounced recovery than group C. The results suggest that the combined treatment approach employed in this study has been optimised, further enhancing its effectiveness compared to prior methods.

### The AI-CS effect on the living quality in patients undergoing laparoscopic cholelithiasis surgery

Quality of life is the most direct evaluation indicator that reflects the effectiveness of patients after treatment. This study evaluated the quality of life through the GIQLI scale, incidence of complications, and survival curve. The data proved that the various indicator scores on the GIQLI scale in patients with E showed a significant increase, and the scores after treatment were higher than those of C. In the analysis of the incidence of complications, there were 2 cases of complications in E, with an incidence rate of only 3.6%, while the incidence rate of complications in C patients was as high as 12.5%, with a meaningful difference. In the survival curve analysis, the overall survival rate of patients with E reached 95% at 3 months after treatment, while the survival rate of C was above 90%, and the survival rate of E was higher than that of C. The evaluation of quality of life shows that combination therapy has a greater prognostic effect on patients than conventional therapy, consistent with previous research results [30].

## Conclusion

In summary, the combination of AI-CS and laparoscopic surgery has a significant therapeutic effect on gallstone patients. Firstly, it can reduce the patient’s stress response, reducing CRP, Ep, Cor, and NE levels. Secondly, it can alleviate inflammation in patients, reduce pain, and improve treatment effectiveness. In addition, combination therapy can promote the recovery of gallbladder function in patients and reduce the CPO, providing a guarantee for rehabilitation. However, this study did not consider the treatment options required for different lesions in gallstone patients. For this purpose, future research will further optimise a combined treatment plan to improve the clinical treatment effect of gallstone patients.

## Dodatak

### Funding

None.

### Author contribution

G. W. designed the study, collected the data, and wrote the manuscript.

### Acknowledgements

None.

### Conflict of interest statement

All the authors declare that they have no conflict of interest in this work.
